# CX3CL1 (fractalkine) and TNFα production by perfused human placental lobules under normoxic and hypoxic conditions in vitro: the importance of CX3CR1 signaling

**DOI:** 10.1007/s00011-013-0687-z

**Published:** 2013-11-24

**Authors:** Dariusz Szukiewicz, Jan Kochanowski, Tarun Kumar Mittal, Michal Pyzlak, Grzegorz Szewczyk, Krzysztof Cendrowski

**Affiliations:** 1 Department of General and Experimental Pathology, Second Faculty of Medicine, Medical University of Warsaw, ul. Krakowskie Przedmiescie 26/28, 00-928 Warsaw, Poland; 2 Department of Neurology, Second Faculty of Medicine, Medical University of Warsaw, ul. Ceglowska 80, 01-809 Warsaw, Poland; 3 Department of Obstetrics and Gynecology, Second Faculty of Medicine, Medical University of Warsaw, ul. Kondratowicza 8, 03-242 Warsaw, Poland

**Keywords:** Fractalkine/CX3CR1, TNFα, Human placenta, In vitro perfusion study, Hypoxia, CX3CR1 expression

## Abstract

**Objective:**

Inflammation and hypoxia activate the fractalkine (CX3CL1) receptor (CX3CR1)-related signaling pathway. Tumor necrosis factor alpha (TNFα) induces CX3CL1, influencing a mechanism of CX3CL1 autoregulation by CX3CR1 expression. We compared spontaneous and lipopolysaccharide (LPS)-induced CX3CL1 and TNFα production by human placenta under normoxic vs. hypoxic conditions, with respect to CX3CR1 expression and its functional status.

**Methods:**

Placental lobules of term placentae (*N* = 24) were perfused extracorporeally. CX3CL1 and TNFα concentrations were measured in the perfusion fluid by ELISA. LPS, anti-CX3CR1 antibodies and pirfenidone were used in respective subgroups. After perfusion, CX3CR1 expression was estimated in placental tissue using quantitative immunohistochemistry, and the final results were adjusted for the mean microvascular density.

**Results:**

The highest increase in CX3CL1 concentration in response to LPS was observed in hypoxia (*p* < 0.05). Unlike in normoxia, anti-CX3CR1 administration in hypoxia significantly reduced the LPS-evoked response. CX3CR1 expression was augmented by hypoxia and reached 260.9 ± 41 (% ±SEM) of the reference value in normoxia. Positive immunostaining for CX3CR1 corresponded to the vascular endothelium. Pirfenidone inhibited hypoxia + LPS-related increase in TNFα production and prevented the up-regulation of CX3CR1.

**Conclusion:**

The modulatory influence of TNFα on CX3CR1 expression in hypoxia and CX3CL1/CX3CR1 interaction may serve as a compensatory mechanism to preserve or augment the pro-inflammatory course of intercellular interactions in placental endothelium.

## Introduction

Because placental vessels lack autonomic innervation, circulating and locally produced humoral factors must play a crucial role in communication between the compartments of the utero-placento-fetal unit [1]. In addition to vascular resistance, almost every function of the mammalian placenta can be controlled and modified by the local cytokine network, which includes the effects of chemokines [2]. Chemokines form a superfamily of cytokines whose major roles involve the modulation of immune response and the guidance of migrating leukocytes towards or away from chemotactic factors, which act as either chemoattractants or chemorepellents, respectively [3]. Depending on the spacing of their two cysteine residues, chemokines can be divided into four groups (subfamilies) [4]. The first papers describing chemokine CX3CL1 (also known as fractalkine or neurotactin) were presented in 1997 by Bazan et al. [5], and Pan et al. [6]. CX3CL1 is encoded on human chromosome 16 and possesses three amino-acid residues between the first two cysteine residues. CX3CL1 is also the lone CX3C(delta) subfamily member [7]. Unlike other chemokines, CX3CL1 is of non-hematopoietic origin and exists in two forms: as a transmembrane protein with the chemokine domain fixed to a long mucin-like stalk and as a soluble peptide released from the cell surface by proteolytic cleavage [8]. The main roles of membrane-bound CX3CL1 include the promotion of leukocyte binding and adhesion and activation of target cells, whereas the soluble chemokine domain of human CX3CL1 is chemotactic for natural killer cells, T cells and monocytes but not neutrophils. This dual function as an adhesive compound and chemoattractant distinguishes CX3CL1 from other chemokines [5, 7].

Data from studies on the role of CX3CL1 in reproduction are still being accumulated. It has been reported that, together with some other cytokines (CCL7, CCL4, CCL14), CX3CL1 is involved in the processes of implantation, invasion of the trophoblast into the spiral uterine arteries, placental angiogenesis, responses to inflammatory and immunological factors in the utero-placental interface and the induction of labor [8–10].

Interestingly, even during the course of normal pregnancy, the immunological status of the placental unit resembles, to some degree, a controlled inflammatory state [11, 12]. Thus, many complications of pregnancy may be related to exaggerated local or systemic inflammatory responses. A successful pregnancy therefore significantly depends on the balance between anti-inflammatory and pro-inflammatory cytokines [12].

CX3CL1 in humans binds to a single Gαi protein-linked transmembrane receptor, CX3CR1 (previously known as V28), to express biological activity [13]. CX3CR1 receptor stimulation leads to the activation of both the CX3CL1-dependent and integrin-dependent migration of cells with augmented adhesion as a result of synergistic reactions [14].

Changes in CX3CR1 expression may be important because autoregulatory interactions between CX3CL1 and CX3CR1 have been reported. It has been proposed that CX3CL1 induces its own expression via the PI3-kinase/PDK1/Akt/NIK/IKK/nuclear factor kappa beta (NF-κB) signaling pathway [15]. Tumor necrosis factor alpha (TNFα) also induces the expression of fractalkine and CX3CR1 in rat aortic smooth muscle cells, and this induction is mediated by NF-κB activation [16].

Many stimuli potentially able to disrupt cell homeostasis, including hypoxia, may induce CX3CL1 secretion [17, 18]. Activation of the CX3CL1/CX3CR1 signaling pathway induces local angiogenesis through two sequential steps: the induction of hypoxia inducible factor 1 alpha (HIF-1α) and vascular endothelial growth factor (VEGF)-A gene expression and subsequent VEGF-A/vascular endothelial growth factor receptor type 2 (VEGFR2 or KDR)-induced angiogenesis [19, 20]. Hypoxia alone, inflammation alone and the coexistence of the two may up-regulate CX3CL1 expression by increasing the local concentrations of CX3CL1 production enhancers, including TNFα, interferon gamma (IFNγ), and interleukin-1 beta (IL-1β). Moreover, hypoxia markedly increases lipopolysaccharide (LPS)-induced TNFα release [21]. Despite these results, some data from both in vitro and in vivo experiments are somewhat contradictory and indicate that hypoxia markedly inhibits the production of CXCL1 by endothelial cells [22, 23].

Endothelial cells of the vascular system, vascular smooth muscle cells and amniotic epithelial cells are the main sources of CX3CL1 in the human placenta and membranes [9, 24]. While the placenta undoubtedly makes an important contribution to the plasma CX3CL1 concentration, synthesis of this chemokine by maternal blood mononuclear cells and other tissues is also likely to be significant, although the exact extent remains uncertain. Our present approach uses a model suitable for assessing the amount of CX3CL1 produced exclusively in the placental vascular compartment, since the blood was replaced by the perfusion fluid.

The aim of this study is to examine spontaneous and LPS-induced CX3CL1 and TNFα production by perfused human placental lobules in vitro with respect to CX3CR1 expression and functional status.

## Materials and methods

### Placental collection

This study was conducted in compliance with international and local laws concerning human experimentation, and the project was approved by the local ethics committee. Heparinized term placentae (*N* = 24; mean gestational age of 277 ± 6 days) were obtained from primigravidas after normal-course pregnancies. The placentae were delivered by elective cesarean sections and were dually perfused in vitro at 37 °C, using a modified Schneider’s method [25, 26]. The indications for cesarean section were high-grade myopia in pregnant woman and breech presentation of the fetus.

Two noncontiguous lobules of similar size were selected and isolated from each placenta for simultaneous perfusion; the first lobule was exposed to normoxic conditions (forming group I), and the other lobule was exposed to hypoxic conditions (forming group II). More detailed clinical characteristics of the two homogenous groups are given in Table 1.

**Table 1:** Clinical characteristics of the two groups studied

Parameter	Group I (normoxic), group II (hypoxic)
Number of patients/newborns/placentas/isolated lobules (*N*)	24/24/24/24 per group
Age of the patients in full years (range; mean; median)	23–31, 26, 27
Parity	0
Gestational age in days (range; mean; median)	271–283, 277, 279
Method of delivery	Cesarean section
Blood pressure during pregnancy	All records within normal range^a^
Proteinuria during pregnancy	Not present
Liver blood tests (aminotransferases, enzymes, AST and ALT levels)	Within normal range^b^
Smoking during pregnancy	None declared active smoking
Diabetes during pregnancy	Not present
Body mass index <21 or >35	None
Mother’s blood (III trimester): hematocrit (Ht), hemoglobin (Hb), red blood cell (RBC) count, mean cell hemoglobin concentration (MCHC)	All within normal ranges^c^
Other identified risk factors	None
Birth weight in grams (range; mean; median)	2,980–3,810, 3,270, 3,250
Sex of newborns (M, male; F, female)	11 M + 13 F
Weight of placenta in grams (range, mean, median)	568–810, 675, 660
Weight of isolated lobule in grams (range, mean, median)	Group I: 87–115, 102; 99/group II: 85–109, 100, 98

Since each placenta was used for the isolation of two similar lobules for group I and II, the groups may be treated as homogenous

^a^The normal range of blood pressure was defined as systolic pressure between 100 and 140 mmHg, and diastolic pressure between 60 and 90 mmHg

^b^The normal range of values for AST is 5–40 units per liter of serum and the normal range of values for ALT is 7–56 units per liter of serum

^c^Hb levels 10.0–13.5 g/dl, RBC count 3.2–4.4 million/μl, MCHC 319–355 g/L, Ht 31–41 %

Immediately after cannulation of the chorionic vessels in the two selected lobules, the placentas were transported on ice to the laboratory in a plastic box filled with cold, sterile phosphate-buffered saline (PBS). The period from detachment of the placenta to the start of perfusion did not exceeded 20 min.

### Placental perfusion and CX3CL1 and TNFα measurements in the perfusion fluid

**Fig. 1:**
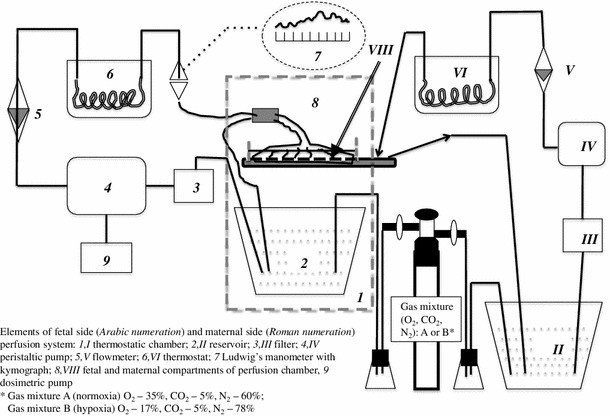
General scheme of in vitro dual placental perfusion system

The single isolated placental lobules (two per placenta) were bilaterally perfused in vitro. A diagram of the two-sided closed perfusion system is shown in Fig. 1. The experimental technique applied in this study has been described in detail in a previous publication [27]. Briefly, the perfusion fluid was isotonic (Ringer–Krebs with antibiotic) and buffered at pH 7.4 (phosphate buffer). The methodological correctness of the in vitro fetal-side placental perfusion process was strictly monitored to maintain perfusion pressure, flow stability, perfusion fluid volume and hydrogen ion concentration (normoxic group only), as shown in Table 2. The perfusion fluid groups were enriched with gas mixtures containing 35 % O_2_, 5 % CO_2_ and 70 % N_2_ (normoxic conditions) or 17 % O_2_, 5 % CO_2_ and 78 % N_2_ (hypoxic conditions). The oxygen concentrations applied in the gas mixtures provided oxygen partial pressures (*p*O_2_) values in the perfusion fluid of 13.3 kPa (normoxic conditions) and *p*O_2_ ≤ 6.5 kPa (hypoxic conditions). These saturation values of dissolved oxygen were monitored during the experimental period by perfusion fluid sampling at 30-min intervals between 30 and 120 min of perfusion. Polarographic (Clark) oxygen electrodes were installed in flow-through thermostatic chambers of both the fetal and the maternal sides of the perfusion system (Fig. 1). This type of *p*O_2_ electrode consists of an anode and cathode and measures oxygen tension amperometrically. It means that the *p*O_2_ electrode produces a current at a constant polarizing voltage (−700 mV) which is directly proportional to the partial pressure of oxygen (*p*O_2_). The current produced is a result of the subsequent reduction of oxygen at the cathode. The oxygen probes [Yellow Springs Instruments (YSI), IL, USA, model 05520-16] delivered the currents to the two-channel biological oxygen monitor (YSI, model 5300A), connected to a computer-assisted data acquisition system.

**Table 2:** Criteria for determining correctness of the in vitro fetal-side placental perfusion

1.	Perfusion pressure: after adaptation phase, pressure should be maintained within 7–11 kPa. Avoidance of rapid changes in blood pressure. Pressure < 7 kPa may be a result of vascular wall rupture. Pressure > 11 kPa suggests occlusion (clot, embolus)
2.	Flow stability: flow velocity 15–20 ml/min may change by no more than ±10 % within 30 s
3.	Perfusion fluid volume: perfusion fluid loss after adaptive phase max. 3 ml/h
4.	pH −log [H+]: after 30, 90 and 150 min of perfusion, pH should not be <7.35, 7.30 and 7.25 in the arterial part of the system, and at least 7.20, 7.15 and 7.10 in the venous part, respectively

Applicable only under normoxic conditions

During the 150 min of perfusion, including the initial 30-min adaptive phase, basal (immediately before administration of LPS) and LPS-evoked (10 ng/ml) CX3CL1 secretion into the fetal side placental circulation was examined quantitatively in perfusion fluid samples using ELISA. The RayBio^®^ Human Fractalkine ELISA Kit (RayBiotech, Inc., USA ) has a very high specificity that exceeds other available ELISA tests for the detection of CX3CL1 in placental perfusates, to the best of our knowledge. The samples were collected every 30 min from 30 to 150 min. The mean values for each group and at each time-point were calculated.

The dose of LPS used in this study was precisely titrated during the preliminary phase of the perfusion experiments. After a series of dose–response curve analyses we established 10 ng/ml as the mean dose of LPS eliciting 45–55 % maximal TNFα response in normoxic conditions (data not shown).

Additionally, the TNFα levels in the perfusion fluid samples obtained at the same time-points were examined. Commercially available kits were used (ELH-TNFalpha-001, RayBio Human TNF-alpha ELISA Kit) following the manufacturer’s instructions. The minimum detectable dose of TNFα was <10 pg/ml.

Experiments also tested the effect of CX3CR1 blockade on CX3CL1 secretion into the fetal side perfusion fluid under normoxic or hypoxic conditions with or without LPS administration. To block CX3CR1 on the fetal side of placental circulation, rabbit anti-human chemokine (C-X3C Motif) receptor 1 (CX3CR1) polyclonal “neutralizing” antibody (ABIN110594; TP502; Torrey Pines Bipolabs, Inc., NJ, USA) was administered via perfusion fluid (fetal side) during the initial adaptive period of the respective perfusions. The final dilution of the antibody amounted to 1 mg/ml and was determined according to available datasheet protocols and experimental measurements. Dilution series were used followed by immunohistochemical staining of the perfused placental tissue specimens for CX3CR1 (see “Immunohistochemistry of CX3CR1”), and comparative (“neutralizing” antibody-treated vs. antibody-free perfusion) quantitative morphometry was used to determine the percentage difference between the stained areas corresponding to CX3CR1 secretion (see “Density of the placental microvessel network”). The difference in expression level for the chosen dilution of 1 mg/ml always exceeded 85 %.

**Fig. 2:**
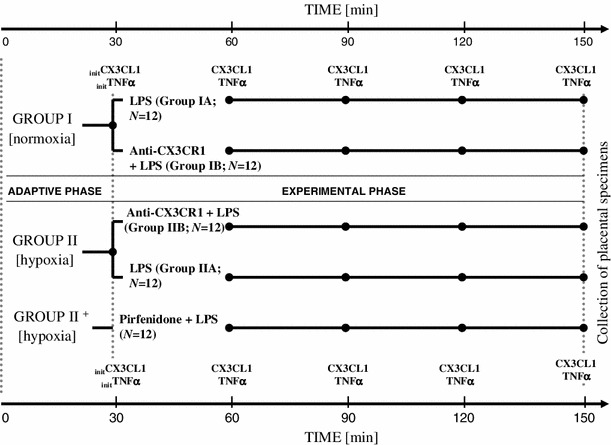
Experimental setup of the perfusion procedures within the groups with the measurement time-points for CX3CL1 and TNFα (*marked with dots*). Initial concentrations of CX3CL1 and TNFα were measured at the end of adaptive phase (_init_CX3CL1 and _init_TNFα, respectively)

The experimental setup for the perfusion procedures and the measurements at fixed time-points is presented in Fig. 2.

Additionally, to investigate the influence of the anti-inflammatory compound pirfenidone, a non-selective inhibitor of TNFα production, another twelve isolated placental lobules (group II^+^) with parameters matching group II were perfused using the same procedure (hypoxic conditions), except that pirfenidone (300 μg/ml; P2116; Sigma-Aldrich Inc., USA) was added into the perfusion fluid.

### Immunohistochemistry of CX3CR1

After completion of the perfusion procedure, two specimens were excised in a standardized manner from each perfused lobule from the region contiguous with the fetal surface of the placenta. After fixation with formalin, six paraffin-embedded 5 μm sections (three for each specimen) were prepared for each examined lobule. The methodology used in the applied in vitro experiment precluded obtaining the placental specimens during the phase of perfusion. Thus, we do not have collected data on CX3CR1 expressions at the same time-points as scheduled for CX3CL1 and TNFα. However, as a kind of control measurement, the tissue specimen of each perfused placental lobule was collected at the end of the adaptive phase for CX3CR1 immunostaining.

To visualize CX3CR1, standard immunohistochemical procedures were used. Rabbit polyclonal antibody IgG to CX3CR1 (ab8020; Abcam Inc., USA; concentration of 10 μg/ml) was used as the primary antibody, and goat anti-rabbit IgG was used as the biotinylated secondary antibody (ab64256; Abcam; 0.5 % v/v). Visualization of the primary anti-receptor antibodies was performed using a StreptABComplex/HRP Duet (Dako Cytomation, Glostrup, Denmark) kit following the procedure recommended by the manufacturer with 3,3′-diaminobenzidine serving as the chromogen. The respective negative controls for immunostaining were prepared simultaneously by replacement of the polyclonal primary antibody with normal rabbit preimmune IgG diluted with PBS containing 3 % bovine serum albumin at the same protein concentration as that for the primary antibody.

### Density of the placental microvessel network

The vessels of the placental sections were identified with rabbit polyclonal antibody anti-CD31 (dilution 1:50, ab28364; Abcam Inc., Cambridge, MA, USA). The tissue was incubated with the primary antibody for 30 min. A biotinylated goat anti-rabbit antibody (Abcam) was used as the secondary antibody. Next, morphometric analysis was performed using light microscopy with a Leica imaging workstation (Quantimet 500C+, Leica), and the vascular/extravascular tissular index (V/EVTI) was estimated in calibrated areas of the placental sections. Each preparation (paraffin section) underwent three area analyses repeated by two independent observers. The single area measured with the picture analysis method totaled 721,320 μm^2^, and the total number of preparations was 144 per group. Measurement of the total vascular area was performed. The total lumen area of all types of identified vessels was summed in both groups. To eliminate technical error caused by uniaxial sectioning of the vessel, the lowest value of Ferret’s diameter was accepted as the diameter for each lumen. Thus, V/EVTI represents the ratio which is most closely correlated with the intensity of vascularization.

### Expression of CX3CR1

After immunostaining, quantitative immunohistochemistry was performed using morphometric software (Quantimet 500C+, Leica, UK) to identify CX3CR1 receptors in 5-μm paraffin-embedded sections of the placental lobule specimens. Light microscopy was used to gather the images. All morphometric procedures were carried out twice by two independent researchers, and the average values were recorded. The intensity of immunostaining was evaluated using the mean color saturation parameter and thresholding in grey-level histograms. Thus, the expression of CX3CR1 corresponded to the total immunostain calibrated area of the sections examined, where color saturation was used as the segmentation criteria for objects. The analyzed image area totaled 138,692 μm^2^ at 200× magnification. In each group, 144 visual fields were analyzed (six visual fields per each isolated lobule). To achieve maximum accuracy of the measurements, the following factors were controlled or monitored: average of image intake, hue, illumination, luminance, power supply, relation of illumination to quantification of the percentage area of positively stained structures, shading correction and warming up. A detailed description of these morphometric procedures has been provided previously [28, 29]. Finally, the results obtained for the specimens examined were adjusted for the mean density of the placental microvessel network. Morphometric results that included the 90 % confidence intervals are reported as the mean percentage values ±SEM.

### Statistical analysis

Mann–Whitney’s *U* test was applied. The results are expressed as the mean ± SEM, medians or mean percentage values ±SEM. The differences between groups I and II (normoxic vs. hypoxic conditions) were deemed statistically significant if *p* < 0.05.

## Results

**Fig. 3:**
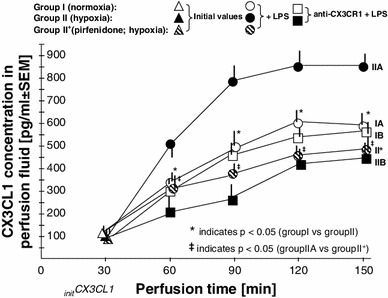
Relationships between the mean CX3CL1 concentration in perfusion fluid after LPS stimulation and CX3CR1 receptor status in hypoxic vs. normoxic conditions. The effect of TNFα inhibitor pirfenidone in hypoxia (group II^+^) is also shown. _*init*_ *CX3CL1*initial CX3CL1 concentration

**Fig. 4:**
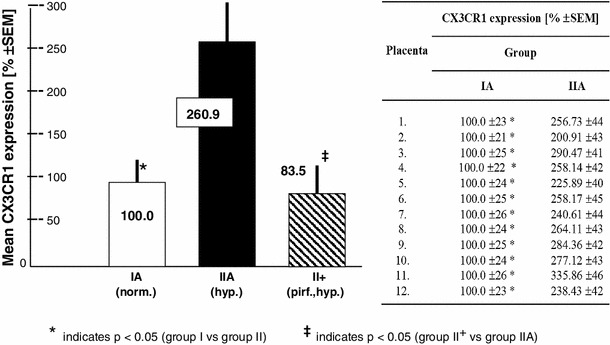
Mean placental expressions of CX3CR1 in normoxia, hypoxia and hypoxia + TNFα inhibitor pirfenidone (groups IA, IIA and II^+^ on the *bar chart*, respectively). The corresponding *table* shows the mean CX3CR1 expression in the same placenta after in vitro perfusion of the two isolated lobules, in normoxia (IA) and hypoxia (IIA). Six visual fields were analyzed for each placental lobule. The mean value in normoxic tissue (group IA) was taken as 100 %

**Fig. 5:**
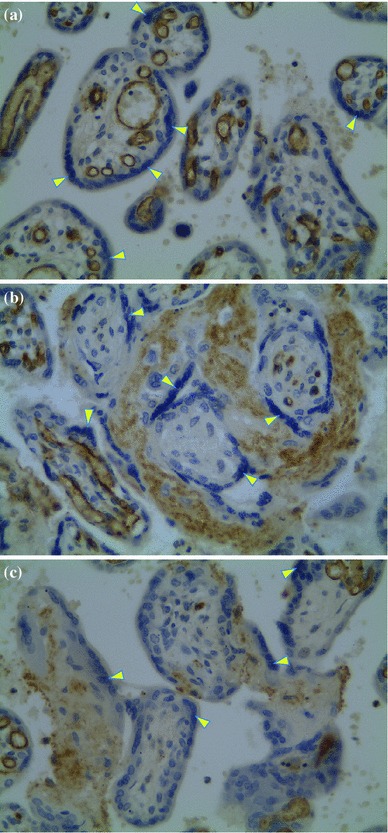
Immunohistochemical visualization of the receptor CX3CR1 in placental tissue at ×400 magnification: **a** normoxia (group IA); **b** hypoxia (group IIA); **c** pirfenidone in hypoxia (group II^+^). Immunostain-positive focal regions correspond to the vascular endothelium (*arrowheads*)

The results pertaining to the CX3CL1 levels are shown in Fig. 3 and Table 3 (see also the experimental setup in Fig. 2). There were no statistically significant differences in the initial concentrations of CX3CL1 (_init_CX3CL1). The mean _init_CX3CL1 in group I was 99.1 ± 27 pg/ml, while group II showed a mean _init_CX3CL1 level of 77.8 ± 33 pg/ml. The median, rounded to the nearest whole number, for group I was 82 [95 % confidence interval (CI) 55–123] pg/ml, whereas in group II, the median was 81 (95 % CI 56–111) pg/ml (Table 3). During the 120-min observation period with specimen collections at four time-points, significant increases in the mean CX3CL1 concentration occurred in all groups studied after the addition of LPS to the perfusion fluid (Table 3; Fig. 3). Compared to group I, the highest CX3CL1 levels (*p* < 0.05) were measured consistently throughout the 120-min perfusion time in hypoxic group IIA. With normoxia, the neutralizing anti-CX3CR1 antibody did not affect the production of CX3CL1. Thus, the differences in the mean CX3CL1 levels between subgroups IA and IB were not statistically significant. Unlike normoxic conditions, blockade of CX3CR1 with hypoxia produced evident disturbance of LPS-induced CX3CL1 release. Despite the increase from its initial values, the mean CX3CL1 concentrations at the respective time-points were lower in group IIB (*p* < 0.05) than in both group IIA and group I. As shown in Fig. 4, the mean expression of CX3CR1 in the placental tissue was significantly augmented in hypoxic group IIA and reached 260.9 ± 41 (% ±SEM) of the reference value obtained in group IA. Positive immunostaining for CX3CR1 in the placenta paraffin sections was found to be localized mainly in areas that corresponded to the vascular endothelium (Fig. 5). The differences between the groups studied in mean CX3CR1 expression at the end of the adaptive phase were not significant (data not showed).

**Table 3:** CX3CL1 concentrations (pg/ml) in the perfusion fluid samples collected at the consecutive time-points

Time-point	Group		
	I (normoxia)	II (hypoxia)	II^+^ (hypoxia): pirfenidone + LPS
Adaptive phase			
30 min (init)			
Mean	99.1 ± 27	77.8 ± 33	65.3 ± 34
Median	82	81	79
Range	55–123	56–111	59–101

Time-point	Group				
	I (normoxia)		II (hypoxia)		II^+^ (hypoxia)
	IA: LPS	IB: anti-CX3CR1 + LPS	IIA: LPS	IIB: anti-CX3CR1 + LPS	Pirfenidone + LPS
Experimental phase					
60 min					
Mean	340.7 ± 35*	300.1 ± 48*	502.9 ± 53^**‡**^	205.2 ± 64	308.8 ± 61
Median	345	311	518	197	305
Range	265–406	227–384	439–600	132–253	249–367
90 min					
Mean	499.6 ± 61*	451.8 ± 52*	778.8 ± 68^**‡**^	258.9 ± 63	382.1 ± 59
Median	486	447	801	222	370
Range	373–571	349–512	667–866	154–294	321–445
120 min					
Mean	602.3 ± 52*	525.4 ± 49*	838.3 ± 76^**‡**^	403.4 ± 51	449.8 ± 45
Median	609	554	842	389	453
Range	495–697	438–593	714–907	324–473	356–521
150 min					
Mean	579.5 ± 54*	550.6 ± 55*	845.6 ± 57^**‡**^	434.2 ± 52	480.5 ± 61
Median	598	548	850	422	465
Range	486–701	470–652	709–913	377–489	384–540

Mean ± SEM, median (rounded to the nearest whole number), and 95 % confidence interval (95 % CI) are shown

*Init* initial concentration

* Indicates *p* < 0.05 (group IA vs. IIA, and group IB vs. IIB)

^**‡**^Indicates *p* < 0.05 (group IIA vs. II^+^)

**Fig. 6:**
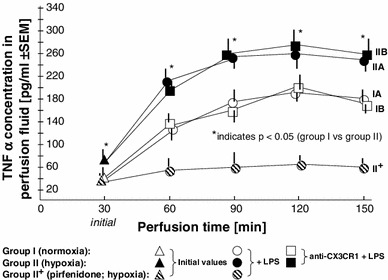
Relationships between the mean TNFα concentration in perfusion fluid after LPS stimulation and CX3CR1 receptor status in hypoxic vs. normoxic conditions. The effect of the TNFα inhibitor pirfenidone in hypoxia (group II^+^) is also shown

As shown in Fig. 6, the initial TNFα concentration (_init_TNFα) measured at the end of the adaptive phase (after the initial 30-min stabilization period) revealed that hypoxia increases the production of this cytokine in perfused placental lobules. The mean _init_TNFα in group I was 39.8 ± 16.8 pg/ml (±SEM), whereas for group II the average _init_TNFα was 72.4 ± 17.1 pg/ml. The medians, rounded to the nearest whole number, were 43 (95 % CI 34–117) pg/ml and 75 (95 % CI 69–160) pg/ml for group I and group II, respectively (Table 4). Moreover, although the levels of TNFα measured in perfusion fluid after LPS administration were not affected by the presence of anti-CX3CR1 in both groups, hypoxic conditions significantly increased the mean TNFα concentrations at all time-points (Fig. 6; Table 4).

**Table 4:** TNFα concentrations (pg/ml) in the perfusion fluid samples collected at the consecutive time-points

Time-point	Group		
	I (normoxia)	II (hypoxia)	II^+^ (hypoxia): pirfenidone + LPS
Adaptive phase			
30 min (init)			
Mean	39.8 ± 16.8*	72.4 ± 17.1	38.1 ± 14.2
Median	43	75	39
Range	34–117	69–160	27–85

Time-poin	Group				
	I (normoxia)		II (hypoxia)		II^+^ (hypoxia)
	IA: LPS	IB: anti-CX3CR1 + LPS	IIA: LPS	IIB: anti-CX3CR1 + LPS	Pirfenidone + LPS
Experimental phase					
60 min					
Mean	121.6 ± 22.2*	133.5 ± 14*	209.3 ± 17.8	192.5 ± 9.6	54.9 ± 17.3
Median	118	127	214	197	56
Range	59–209	63–229	98–287	106–290	46–107
90 min					
Mean	176.6 ± 24.7*	156.1 ± 19.5*	250.5 ± 6.6	252.5 ± 19.7	59.3 ± 21.2
Median	181	153	257	258	61
Range	106–291	87–273	140–369	128–346	49–113
120 min					
Mean	184 ± 16.3*	196.3 ± 23.8*	258.7 ± 18.3	271.9 ± 19.7	64.5 ± 7.5
Median	178	191	263	269	58
Range	111–298	109–345	136–377	158–405	38–129
150 min					
Mean	179.4 ± 23.2*	170.2 ± 11.7*	243.8 ± 9.2	252.8 ± 19.9	60.4 ± 8.9
Median	183	173	242	260	61
Range	123–275	130–283	157–411	149–424	42–124

Mean ± SEM, median (rounded to the nearest whole number), and 95 % confidence interval (95 % CI) are shown

*Init* initial concentration

* Indicates *p* < 0.05 (group IA vs. group IIA, and IB vs. group IIB)

As shown in Fig. 6 and Table 4, the presence of pirfenidone in the perfusion fluid almost completely inhibited hypoxia and the LPS-related increase in the TNFα concentration. Pirfenidone also prevented the up-regulation of CX3CR1 (Fig. 4).

## Discussion

This study increases the number of scientific approaches that have been used to understand the role of locally produced cytokines, including chemokines, in placental homeostasis [8, 11, 24]. We have demonstrated the modulatory influence of the cytokine TNFα in hypoxia on an autoregulatory mechanism through the interaction of chemokine CX3CL1 with its receptor CX3CR1 in human placental circulation.

The results obtained are strongly influenced by the experimental model chosen in our study. First, the circulating blood in the vascular system of the isolated lobules was replaced with perfusion fluid. The elimination of blood removed a rich source of cytokines and produced the opportunity for a more selective and simple interpretation of the observed relationships, at the endothelial cell level [18]. In contrast, the lack of circulating cytokines and activated leukocytes in the blood may be a disadvantage and reduces the likelihood of direct implementation of the results in clinical studies [30, 31].

Local hypoxemia/hypoxia and its accompanying lowered pH significantly affected the cytokine profiles that have been typically observed in placental infections or pre-eclampsia as well as in many cases of fetal intrauterine hypotrophy syndrome or intrauterine growth retardation [32–34]. Lowered oxygen content, however, especially during placental development in the first trimester, should also be considered a physiological modulator of angiogenesis [32].

There is a general consensus of opinion among independent scientists that both hypoxia and inflammatory factors (LPS) may significantly increase the production of TNFα by endothelial cells, including the placental endothelium [2, 32, 35]. Together with a wide array of inflammatory stimuli, such as LPS, IL-1, IFNγ and VEGF-A/KDR, TNFα is able to enhance the expression and production of CX3CL1 in endothelial cells [18, 22]. A post-transcriptional regulatory mechanism was proposed to explain the synergistic induction of CX3CL1 expression by TNFα and IFNγ. In this mechanism, TNFα activates the pathway which involves the stabilization of CX3CL1 mRNA mediated by p38 MAPK/MAPK-activated protein kinase-2 activation [36].

According to our results, there is no reason to doubt that LPS and TNFα augment the production of chemokine CX3CL1. Similar results, including but not limited to LPS and TNFα production, were observed by other investigators in research concerning inflammatory cytokine signaling in endothelial cells [18, 22, 35]. However, there is a discrepancy between the available data for CX3CL1 expression and production in hypoxic environments. Some authors have suggested that hypoxia-induced oxidative stress stimulates CX3CL1, while others have reported the opposite effect [18, 22, 37]. For example, in a study conducted using human umbilical vein endothelial cells, hypoxia suppressed IFNγ-induced CX3CL1 expression [22]. We have demonstrated a significant increase in CX3CL1 concentration in the perfusion fluid under hypoxia, which is linked with the up-regulated expression of CX3CR1 in the endothelial compartment of placental tissue.

These contradictory results have created uncertainty regarding whether endothelium-derived CX3CL1 acts in an organ-, site- or state-dependent manner. Recent data indicate that chemokines have various effects on endothelial cells depending on their organ of origin [38, 39]. Moreover, it has been proposed that these activities, such as proliferation, lymphocyte adhesion and angiogenesis, are not correlated with receptor expression [39].

The mechanisms contributing to increased expression of CX3CR1 in hypoxic conditions may involve HIF-1-dependent and NF-κB-dependent signaling pathways, as shown in prostate cancer cells [40]. Furthermore, the autoregulatory mechanism through which CX3CL1 induces its own expression includes NF-κB-inducing kinase (NIK), inhibitory κB (IκB) kinase (IKK), NF-κB, Akt, phosphoinositide 3-kinase (PI 3-kinase), and phosphoinositide-dependent kinase 1 (PDK1) [15, 41]. Overexpression of CX3CR1 in response to hypoxia may serve as another autoregulatory and compensatory mechanism to preserve or augment the pro-inflammatory course of intercellular interactions within the local (e.g., placental) cytokine network [15, 18]. As observed in our study, “neutralization” of these receptors by anti-CX3CR1 in hypoxia resulted in the deterioration of the ability to increase CX3CL1 production through this mechanism.

CX3CL1 might play an important role in the pathophysiological process of inflammatory angiogenesis. The angiogenic activity of CX3CL1 required two signal pathways: RAF-1/MEK/ERK and PI3K/Akt/eNOS/NO. Interestingly, both pathways were mediated via CX3CR1 [42].

In endothelial cells and other cell types, TNFα acts through NF-κB to strongly induce both CX3CR1 expression and CX3CL1 production [15, 43]. Pirfenidone, a non-selective inhibitor of inflammatory mediators including TNFα, was consistently able to almost eliminate increases in CX3CL1 levels in the hypoxic perfusion fluid (group II^+^) and counteracted CX3CR1 overexpression.

In conclusion, these results indicate that, in hypoxia, interaction between the two important members of the placental cytokine network, TNFα and CX3CL1, may be influenced by increased TNFα levels. Up-regulation of CX3CR1 is needed for increased CX3CL1 production in hypoxia. TNFα-induced up-regulation of CX3CR1 expression is crucial and most likely overrides CX3CL1 autoregulation. Further studies on an in vivo placental perfusion model with blood-filled vessels is required for more definite conclusions, including potential treatment options in selected hypoxia- and hypoxemia-related inflammatory gestational pathologies.

## References

[1] Myatt L (1992). Control of vascular resistance in the human placenta. Placenta.

[2] Hauguel-de Mouzon S, Guerre-Millo M (2006). The placenta cytokine network and inflammatory signals. Placenta.

[3] Steinke JW, Borish L (2006). Cytokines and chemokines. J Allergy Clin Immunol.

[4] Raman D, Sobolik-Delmaire T, Richmond A (2011). Chemokines in health and disease. Exp Cell Res.

[5] Bazan JF, Bacon KB, Hardiman G, Wang W, Soo K, Rossi D (1997). A new class of membrane-bound chemokine with a CX3C motif. Nature.

[6] Pan Y, Lloyd C, Zhou H, Dolich S, Deeds J, Gonzalo JA (1997). Neurotactin, a membrane-anchored chemokine upregulated brain inflammation. Nature.

[7] Stievano L, Piovan E, Amadori A (2004). C and CX3C chemokines: cell sources and physiopathological implications. Crit Rev Immunol.

[8] Bowen JM, Chamley L, Mitchell MD, Keelan JA (2002). Cytokines of the placenta and extra-placental membranes: biosynthesis, secretion and roles in establishment of pregnancy in women. Placenta.

[9] Hannan NJ, Jones RL, White CA, Salamonsen LA (2006). The chemokines, CX3CL1, CCL14, and CCL4, promote human trophoblast migration at the feto-maternal interface. Biol Reprod.

[10] Hannan NJ, Salamonsen LA (2008). CX3CL1 and CCL14 regulate extracellular matrix and adhesion molecules in the trophoblast: potential roles in human embryo implantation. Biol Reprod.

[11] Yip L, McCluskey J, Sinclair R (2006). Immunological aspects of pregnancy. Clin Dermatol.

[12] Hunt JS (2006). Stranger in a strange land. Immunol Rev.

[13] Imai T, Hieshima K, Haskell C, Baba M, Nagira M, Nishimura M (1997). Identification and molecular characterization of fractalkine receptor CX3CR1, which mediates both leukocyte migration and adhesion. Cell.

[14] Dogrell SA (2011). CX3CR1 as a target for airways inflammation. Expert Opin Ther Targets.

[15] Chandrasekar B, Mummidi S, Perla RP, Bysani S, Dulin NO, Liu F (2003). Fractalkine (CX3CL1) stimulated by nuclear factor kappa B (NF-kappaB)-dependent inflammatory signals induces aortic smooth muscle cell proliferation through an autocrine pathway. Biochem J.

[16] Sung MJ, Kim DH, Davaatseren M, Hur HJ, Kim W, Jung YJ (2010). Genistein suppression of TNF-alpha-induced fractalkine expression in endothelial cells. Cell Physiol Biochem.

[17] Kim KW, Vallon-Eberhard A, Zigmond E, Farache J, Shezen E, Shakhar G (2011). In vivo structure/function and expression analysis of the CX3C chemokine fractalkine. Blood.

[18] Imaizumi T, Yoshida H, Satoh K (2004). Regulation of CX3CL1/fractalkine expression in endothelial cells. J Atheroscler Thromb.

[19] Ryu J, Lee CW, Hong KH, Shin JA, Lim SH, Park CS (2008). Activation of fractalkine/CX3CR1 by vascular endothelial cells induces angiogenesis through VEGF-A/KDR and reverses hindlimb ischaemia. Cardiovasc Res.

[20] Mehard B, Keane MP, Strieter RM (2007). Chemokines as mediators of angiogenesis. Thromb Haemost.

[21] Hempel SL, Monick MM, Hunninghake GW (1996). Effect of hypoxia on release of IL-1 and TNF by human alveolar macrophages. Am J Respir Cell Mol Biol.

[22] Yamashita K, Imaizumi T, Hatakeyama M, Tamo W, Kimura D, Kumagai M (2003). Effect of hypoxia on the expression of fractalkine in human endothelial cells. Tohoku J Exp Med.

[23] van Meurs M, Wulfert FM, Jongman RM, Schipper M, Houwertjes MC, Vaneker M (2011). Hemorrhagic shock-induced endothelial cell activation in a spontaneous breathing and a mechanical ventilation hemorrhagic shock model is induced by a proinflammatory response and not by hypoxia. Anesthesiology.

[24] Shimoya K, Zhang Q, Tenma K, Ota Y, Hashimoto K, Shizusawa Y (2003). Fractalkine (FRK) levels in amniotic fluid and its production during pregnancy. Mol Hum Reprod.

[25] Schneider H, Challier JC, Dancis J (1981). Transfer and metabolism of glucose and lactate in the human placenta studied by a perfusion system in vitro. Placenta.

[26] Szukiewicz D, Szukiewicz A, Maslinska D, Markowski M (1999). In vitro effect of bioactive natriuretic peptides on perfusion pressure in placentas from normal and pre-eclamptic pregnancies. Arch Gynecol Obstet.

[27] Szukiewicz D, Maslinska D, Gujski M, Pyzlak M, Klimkiewicz J, Stelmachow J (2008). Angiotensin II (Ang II) evoked secretion of the human placental lactogen (HPL) in intrauterine growth retardation: examination of the relationship with Ang II receptor type 1 (AT1) expression. Int Immunopharmacol.

[28] Szukiewicz D, Gujski M, Maslinska D, Szewczyk G, Bachanek M, Maslinski S (2005). Mast cell-derived VEGF and VEGF receptor type 1, 2 and 3 expression in human term trophoblast culture—influence of hypoxia. Inflamm Res.

[29] Huppertz B, Abe E, Murthi P, Nagamatsu T, Szukiewicz D, Salafia C (2007). Placental angiogenesis, maternal and fetal vessels—a workshop report. Placenta.

[30] Kraus TA, Sperling RS, Engel SM, Lo Y, Kellerman L, Singh T (2010). Peripheral blood cytokine profiling during pregnancy and post-partum periods. Am J Reprod Immunol.

[31] Umehara H, Bloom ET, Okazaki T, Nagano Y, Yoshie O, Imai T (2004). Fractalkine in vascular biology: from basic research to clinical disease. Arterioscler Thromb Vasc Biol.

[32] Cartwright JE, Keogh RJ, Tissot van Patot MC (2007). Hypoxia and placental remodeling. Adv Exp Med Biol.

[33] Keelan JA, Mitchell MD (2007). Placental cytokines and preeclampsia. Front Biosci.

[34] Coussons-Read ME, Lobel M, Carey JC, Kreither MO, D’Anna K, Argys L (2012). The occurrence of preterm delivery is linked to pregnancy-specific distress and elevated inflammatory markers across gestation. Brain Behav Immun.

[35] Kataoka T (2009). Chemical biology of inflammatory cytokine signaling. J Antibiot (Tokyo).

[36] Matsumiya T, Ota K, Imaizumi T, Yoshida H, Kimura H, Satoh K (2010). Characterization of synergistic induction of CX3CL1/fractalkine by TNF-alpha and IFN-gamma in vascular endothelial cells: an essential role for TNF-alpha in post-transcriptional regulation of CX3CL1. J Immunol.

[37] Zhang J, Hu H, Palma NL, Harrison JK, Mubarak KK, Carrie RD (2012). Hypoxia-induced endothelial CX3CL1 triggers lung smooth muscle cell phenotypic switching and proliferative expansion. Am J Physiol Lung Cell Mol Physiol.

[38] Walenta KL, Bettink S, Böhm M, Friedrich EB (2011). Differential chemokine receptor expression regulates functional specialization of endothelial progenitor cell subpopulations. Basic Res Cardiol.

[39] Crola Da Silva C, Lamerant-Fayel N, Paprocka M, Mitterrand M, Gosset D, Dus D (2009). Selective human endothelial cell activation by chemokines as a guide to cell homing. Immunology.

[40] Xiao LJ, Chen YY, Lin P, Zou HF, Zhao LN, Li D (2012). Hypoxia increases CX3CR1 expression via HIF-1 and NF-κB in androgen-independent prostate cancer cells. Int J Oncol.

[41] Kasama T, Wakabayashi K, Sato M, Takahashi R, Isozaki T (2010). Relevance of the CX3CL1/fractalkine-CX3CR1 pathway in vasculitis and vasculopathy. Transl Res.

[42] Lee SJ, Namkoong S, Kim YM, Kim CK, Lee H, Ha KS (2006). Fractalkine stimulates angiogenesis by activating the Raf-1/MEK/ERK- and PI3K/Akt/eNOS-dependent signal pathways. Am J Physiol Heart Circ Physiol.

[43] Ahn SY, Cho CH, Park KG, Lee HJ, Lee S, Park SK (2004). Tumor necrosis factor-alpha induces fractalkine expression preferentially in arterial endothelial cells and mithramycin A suppresses TNF-alpha-induced fractalkine expression. Am J Pathol.

